# Sex-dependent effects of microcystin-LR on hypothalamic-pituitary-gonad axis and gametogenesis of adult zebrafish

**DOI:** 10.1038/srep22819

**Published:** 2016-03-10

**Authors:** Wanjing Liu, Chuanyue Chen, Liang Chen, Li Wang, Jian Li, Yuanyuan Chen, Jienan Jin, Atufa Kawan, Xuezhen Zhang

**Affiliations:** 1 College of Fisheries, Huazhong Agricultural University, Freshwater Aquaculture Collaborative Innovation Center of Hubei Province, Wuhan 430070, People’s Republic of China; 2 Donghu Experimental Station of Lake Ecosystems, State Key Laboratory of Freshwater Ecology and Biotechnology, Institute of Hydrobiology, Chinese Academy of Sciences, Wuhan 430072, People’s Republic of China

## Abstract

While microcystins (MCs) have been reported to exert reproductive toxicity on fish with a sex-dependent effect, the underlying mechanism has been rarely investigated. In the present study, zebrafish were exposed to 1, 5 and 20 μg/L MC-LR for 30 d. The gonad-somatic index declined in all treated males. 17β-estradiol (E_2_), testosterone (T), 11-keto testosterone (11-KT) and follicle-stimulating hormone (FSH) levels increased in serum from all treated females, while T, FSH and luteinizing hormone (LH) levels changed in all treated males. Histomorphological observation showed that MC-LR exposure evidently retarded oogenesis and spermatogenesis. Transcriptional changes of 22 genes of the hypothalamic-pituitary-gonad (HPG) axis exhibited sex-specific responses, and the relationship between gene transcriptions and gametogenesis was evaluated by principle component analysis (PCA). Major contributors to PC1 (*gnrh2, gnrhr3, ar, lhr, hmgra, hmgrb and cyp19a*) were positively correlated with the number of post-vitellogenic oocytes, while PC1 (*gnrh2, lhβ, erβ, fshr, cyp11a and* 17*βhsd*) were positively correlated with the number of spermatozoa. The protein levels of 17βHSD and CYP19a were affected in both females and males. In conclusion, this study first investigated the sex-dependent effects of microcystins on fish reproduction and revealed some important molecular biomarkers related to gametogenesis in zebrafish suffered from MC-LR.

Cyanobacteria are photosynthetic, oxygen producing, gram-negative microorganisms found ubiquitously in nature even at extreme climatic conditions^1^,^2^. Increasing global temperature, nutrient and pollutant enrichment via anthropogenic runoff, drought and flooding lead to eutrophication and outbreak of cyanobacterial blooms^3^,^4^. The toxic cyanobacterial blooms can produce and release cyanotoxins, the secondary metabolites of cyanobacteria, into water^5^. Among all the cyanobacterial toxins, microcystins (MCs) represent a family of potent hepatotoxins and are considered as the most resistant of cyanotoxins to degradation because of their stable cyclic peptide structure^6^. To date, over 100 variants of MCs have been isolated, and microcystin-LR (MC-LR), a hydrophobic variant, is considered to be the most commonly occurring and lethal toxin^7^. In fish, MCs accumulate mainly in liver and therefore liver is considered as the first target organ of MCs^8^,^9^. A provisional safety guideline of 1.0 μg/L MC-LR in drinking water was recommended by the World Health Organization (WHO)^10^.

MCs are released from the cyanobacterial cells into the water bodies where fish spends their whole life stage, including growth, reproduction and embryonic development^11^. Gonad has been recognized as the second important target organ of MCs^12^ and MCs exert negative effects on the reproductive system of fish^13^,^14^,^15^. Lysis of the gonadosomatic tissue in ovary and disruption of spermatogenesis in testis were observed in medaka fish exposed to MC-LR^13^. Hou *et al*. also suggested MC-LR had adverse effects on histological structure and functional activity of the gonads in female zebrafish^15^. Our previous study put forward the idea that female zebrafish were more vulnerable than males to MCs exposure^16^. Deng *et al*. also demonstrated that dietary MC-LR affected reproduction performance in medaka and the effects were gender dependent^17^. However, the potential molecular mechanisms for sex-dependent reproductive toxicity of micorcystins on fish are still relatively insufficiently known.

Endocrine disrupting effects have been reported in larval zebrafish exposed to *Microcystins*^18^ and crucian carp exposed to extracted microcystins^19^. Many studies have also demonstrated that MCs can disrupt the synthesis of steroid hormones resulting in dysfunction of endocrine system and reproductive toxicity^7^,^16^,^20^. Oziol and Bouaïcha confirmed MC-LR could act as an endocrine disruptor (ED) in cultured mammalian cells by indirect interaction with estrogen receptors at concentrations lower than those found in natural environments^21^. Reproductive processes in fish are regulated by coordinated interactions among steroid hormones along the hypothalamic-pituitary-gonad (HPG) axis and by steroidogenesis of gonad tissues^22^. A significant degree of evolutionary conservation has been found to occur in the basic aspects of the HPG axis among vertebrates^23^. Therefore, the HPG model serves as a reasonable system and critical tool for conducting ecotoxicological studies focused on extrapolation across species^24^,^25^ and has been applied to predict risks associated with chemical exposure^26^,^27^,^28^. Gonadotropin-releasing hormones (GnRHs) released from the hypothalamus stimulate secretion of gonadotropin hormones (GtHs), including follicle-stimulating hormone (FSH) and luteinizing hormone (LH), from the pituitary into the blood. GtHs are then transported to the gonads to induce steroidogenesis producing sex steroid hormones, such as 17β-estradiol (E_2_) and testosterone (T), which modulate the reproductive process^29^. Some studies have reported that some EDs affected the HPG axis in fish in a sex-dependent way^26^,^28^. What is the potential mechanism of the sex-dependent toxicity induced by MC in fish? We suppose that the gene networks of HPG axis might play an important regulatory role.

In our previous studies, we demonstrated that MC-LR exerted sex-dependent reproductive toxicity on adult zebrafish^16^, and developmental toxicity on F1 offspring as a consequence of disruption of oogenesis and spermatogenesis in parental zebrafish^30^. However, further studies were required to reveal the underlying molecular mechanism of the sex-specific effects. We, therefore, investigated the endocrine disrupting effects of MC-LR on the HPG axis and gametogenesis in zebrafish. Using principle component analysis (PCA) we found some sex-specific genes used as biomarkers for gametogenesis suppression caused by microcystins or other EDs.

## Material and Methods

### Toxin and Chemicals

1 mg MC-LR was purchased from Taiwan Algal Science Inc. with a purity of 95% and was dissolved in 1 mL milliQ water to obtain a concentration of 1 mg/mL. To measure actual concentrations of the exposure media, water samples were collected from each tank for 6 times during the 30 d exposure period and determined through a commercially available MC plate kit (Beacon Analytical Systems, Inc., Saco, ME, USA) (Supporting Information Table S1). The detection limit for MC was 0.1 μg/L. All other reagents bought from multifarious commercial sources were of analytical grade.

### Fish Maintenance, Exposure and Sampling

Adult zebrafish (AB-type, 4 mo old) were obtained from zebrafish breeding center in Institute of Hydrobiology, Chinese Academy of Sciences and acclimated for approximately 14 d in a temperature controlled room (28 ± 1 °C). Male and female fish were separately cultured in glass tanks with filtered tap water under a photoperiod of 14:10 h light:dark and fed three times a day with *Artemia nauplii* (<24 h after hatching). Water quality parameters, such as pH, temperature and dissolved oxygen, were measured weekly.

After the acclimation period, males and females were exposed to control (cultured water), 1, 5 or 20 μg/L of MC-LR for 30 d. The dosages and exposure time of MC-LR were designed based on our previous work on reproductive toxicity of MCs^16^,^30^. In each group, the treatment was performed in three different aquaria (5 L exposure medium, 10 fish each aquarium). Every 3 d one-third of the treated water was renewed with freshly prepared solution containing the corresponding MC-LR or no MC-LR. No mortality was observed at any treatment during the exposure period.

After the exposure, fish were anesthetized by submersion in 0.1 g/L 3-aminobenzoic acid ethyl ester (MS-222), and total weight and length were recorded for each fish. Indices including condition factor (K = weight (g)/snout−vent length (cm)^3^ × 100), brain-somatic index (BSI = brain weight × 100/body weight) and gonad-somatic index (GSI = gonad weight × 100/body weight) were calculated. The experiment was approved by the guidelines of Institutional Animal and Care and Use Committees (IACUC) of Huazhong Agricultural University, Wuhan of China, and the methods were carried out in accordance with the approved guidelines.

### Histopathological Observation

After MC-LR exposure, adult females (n = 3) and males (n = 3) from each experimental group were sacrificed. For light microscopy, the samples of gonads were first fixed in 10% buffered formalin for 24 h at 4 °C, and then immediately dehydrated in graded series of ethanol, immersed in xylol and embedded in paraffin wax. Sections of 4 μm were mounted. After deparaffinized, the sections were rehydrated, stained with hematoxylin and eosin. They were examined and photographed under photon microscope.

### Oogenesis and Spermatogenesis

The ovary of zebrafish is in fact asynchronous and oocytes at different stages of development are simultaneously present^28^. The oocytes could be divided into three different groups according to their sizes: previtellogenic (0.15–0.34 mm Ø), vitellogenic (0.35–0.69 mm Ø) and postvitellogenic (0.70–0.75 mm Ø)^31^. Each follicle stage was expressed as a percent of the total number (5000) of follicles from both ovaries of each female used. In the testis of zebrafish, three major stages of spermatogenesis were analyzed: spermatogonia, spermatocytes or spermatids^32^ and the percentage of cells at each stage was calculated from 10000 cells per male zebrafish.

### Hormone Measurement

After exposure, blood sample was collected from caudal vein of the fish using a glass capillary tube. Between five and eight of blood per each fish was centrifuged at 3,000 × g for 20 min at 4 °C, and the serum was stored at −80 °C. Plasma sex steroid hormones E_2_, T and 11-keto testosterone (11-KT) were measured by using competitive enzyme-linked immunosorbent assay (ELISA) kits (E_2_ (Item No. 582251), T (Item No. 582701), 11-KT (Item No. 582751); Cayman Chemical Company, Ann Arbor, Mi, USA), following the manufacturer’s instructions. The plasma hormones FSH and LH were detected by using ELISA kits (Shanghai Enzyme-linked Biotechnology Company, Shanghai, China) according to the manufacturer’s instructions.

### Real-Time Polymerase Chain Reaction (PCR) Assay

Samples of brain and gonad of each fish were collected after 30 d of exposure and were measured for transcriptions of 22 genes representing key signaling pathways and functional processes of the HPG axis (For the full name of the genes and the sequences of primers for the genes measured, refer to Table 1 and Supporting Information Table S2). Samples of each organ were preserved in Sample Protector (TaKaRa, Dalian, China) at −80 °C until analysis. Total RNA was isolated from the sample by use of RNAiso Plus (TaKaRa, Dalian, China) and detected by an ultraviolet spectrophotometer and agarose electrophoresis. For each sample, 1 μg total RNA used for reverse transcription by tuse of the PrimeScript^®^ RT reagent Kit with gDNA Eraser (TaKaRa, Dalian, China). Quantitative real-time PCR (qRT-PCR) was performed to detect the expression patterns of all target genes. PCR reaction mixtures (20 μL total volume) contained 10 μL of 2 × iTaq universal SYBR Green supermix (Bio-Rad, Hercules, CA, USA), 0.50 μM of forward and reverse primers each and 0.1 μg of cDNA sample. Samples were pre-denatured at 95 °C for 5 min, followed by 40 cycles of denaturation at 95 °C for 5 s, annealing at 55–60 °C for 20 s and elongation at 72 °C for 20 s. Each plate contained a no template control with water instead of the template. For quantification of PCR results, the threshold cycle (Ct) was determined for each reaction. Using GeNorm analysis^33^, the results demonstrated that *β-actin* was the most stable gene among the four commonly used housekeeping genes *glyceraldehydes-3-phosphate dehydrogenase* (*gapdh*), *elongation factor 1-alpha* (*ef1α*), *18s ribosomal RNA* (*18s rRNA*) and *β-actin* in both male and female brain and gonad. Ct values for each gene of interest were normalized to the endogenous control gene, *β-actin*, by using the ^ΔΔ^Ct method^34^. Normalized values were used to detect the degree of inhibition or induction expressed as a “fold difference” compared to normalized control values. For each group, six samples were measured.

### Western Blot Analysis

Western blot analysis was performed to determine the expression of 17βHSD and CYP19a proteins in gonads of female and male zebrafish exposed to MC-LR. Western blot analysis was conducted using standard methods with modification^35^. Briefly, tissue samples were homogenized in RIPA lysis buffer (Beyotime, China) containing protease inhibitor at 4 °C, and centrifuged at 12,000 × g for 15 min. The supernatant was aliquoted and protein concentration was determined by BCA kit (Beyotime, China). Thirty microgram total proteins of each sample were separated by electrophoresis in 10% SDS-PAGE gels. After electrophoresis, proteins were transferred onto PVDF membranes (Millipore, Massachusetts, USA) at 200 mA for 2 h at 4 °C. Subsequently, the membranes were blocked with TBST (20 mmol/L Tris, 500 mmol/L NaCl and 0.05% Tween 20) containing 5% skim milk power for 2 h at room temperature, then incubated with anti-17βHSD antibody (GTX48613, GeneTex, USA, 1:500 dilution) or anti-CYP19a antibody (GTX54199, GeneTex, USA, 1:500 dilution) overnight at 4 °C. Anti-GAPDH antibody (Ab181602, Abcam, UK, 1:10000 dilution) was selected as internal reference (Supporting Information Table S3). After primary antibody incubation, the membranes were washed with TBST and incubated in goat anti-rabbit secondary antibody (074–1506, KPL, USA, 1:10000 dilution) for 2 h at room temperature. Immunoreactivity was visualized by colorimetric reaction using ECL substrate buffer (Tuojie, Wuhan, China). Membranes were scanned with Gel Doz EZ imager (BIO-RAD, USA).

### Data Analysis

Normality and homogeneity of data were evaluated by using the Kolmogorov-Smirnow and Levene’s tests, respectively. The statistical differences of the experimental data were evaluated by Dunnett’s one-way analysis of variance (ANOVA) using SPSS software package.

To identify which genes were responsible for the differences detected in germ cell number, the following statistics were performed. Spearman correlation analysis was conducted independently for females and males using SPSS, to determine the bivariate correlation between 22 gene mRNA levels in the HPG axis. Principle component analysis (PCA) was performed to transform a number of potentially correlated independent variables (gene transcriptions) into a smaller number of orthogonal variables called “principal components (PCs)” using SPSS. With the first two PCs, we assessed the relationship between gene transcriptions and gametogenesis by regression analysis. The linear regression was also applied to evaluate the relationship between gametogenesis and hormone levels.

Statistical differences were determined as *P* < 0.05 and *P* < 0.01 levels for all analyses and indicated with * and **, respectively. All results were expressed as mean ± standard error (S.E.).

## Results

### Organism Level Changes

**Figure 1:**
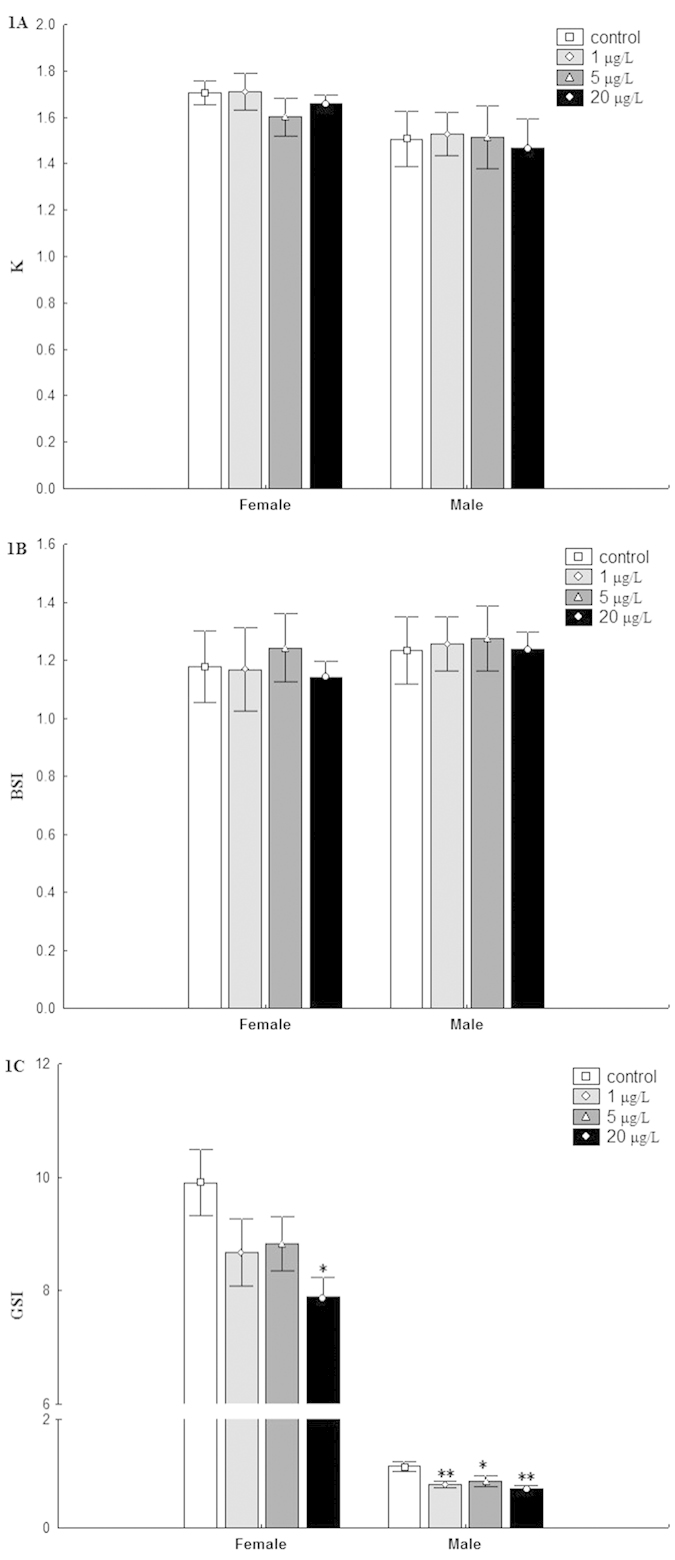
Effects on somatic indices of zebrafish after 30 d exposure to MC-LR. K = weight (g)/snout−vent length (cm)^3^ × 100. BSI = brain weight × 100/body weight. GSI = gonad weight × 100/body weight (mean ± S.E., n = 5). Asterisk (*) and (**) indicate significant differences at *P* < 0.05 and *P* < 0.01 between MC-LR treated groups and the control group, respectively.

The effects of MC-LR on K, BSI and GSI of adult zebrafish are summarized in Fig. 1. The exposure concentrations used in the present study did not result in marked effects on K and BSI in both female and male fish. The GSI of males was dramatically decreased in all treatments (*P* < 0.05), while a significant decrease were only observed in females exposed to 20 μg/L MC-LR.

### Histopathological Observation of Gonads

**Figure 2:**
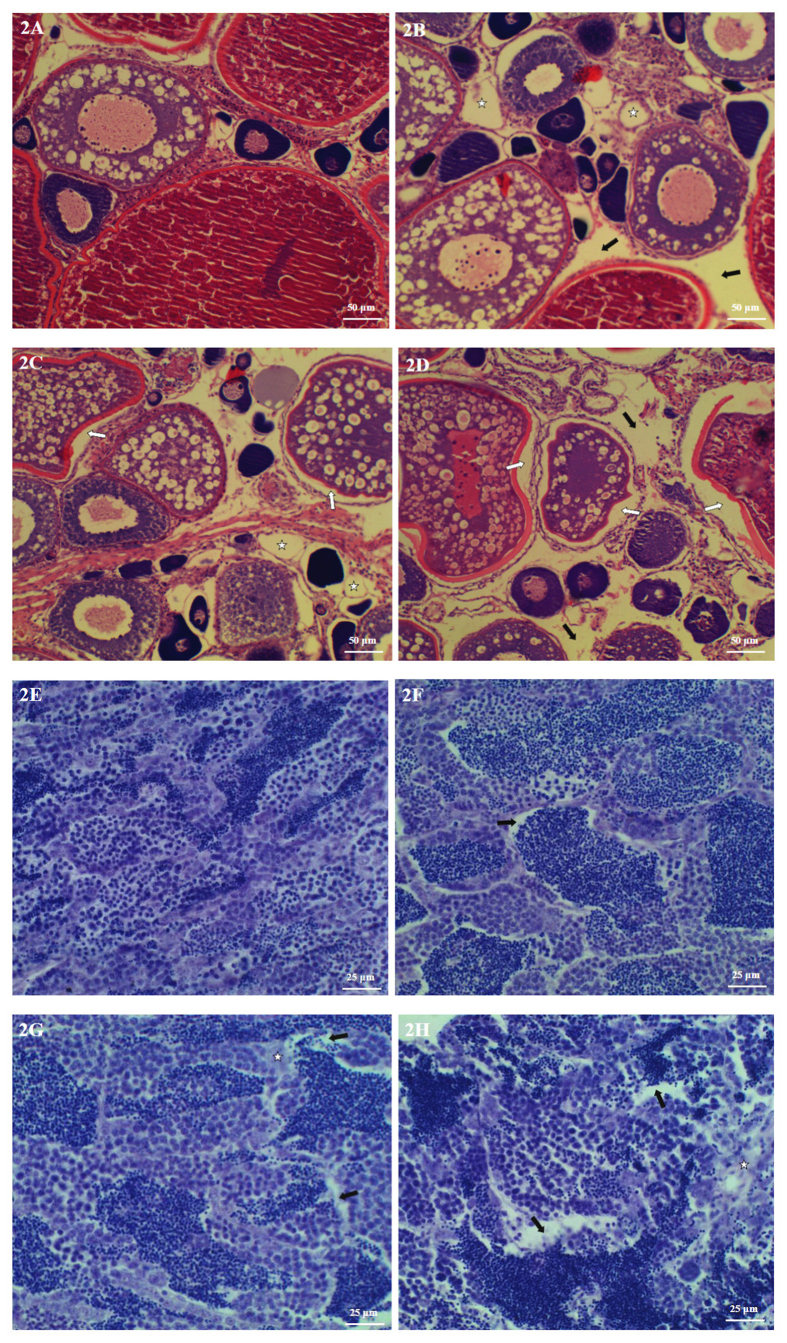
Representative photomicrographs demonstrating lesions in ovary (**A**–**D**) and testis (**E**–**H**) of zebrafish exposed to MC-LR for 30 d. (**A**–**D**) H.E-stained ovary section. Control (**A**); 1 μg/L exposure (**B**): the vacuolation of the gonadosomatic tissue (star), widened intercellular space of the oocyte (black arrow); 5 μg/L exposure (**C**): the vacuolation of the gonadosomatic tissue (star), loss of contact between the oocyte cell membranes and the follicular cell layer (white arrow); 20 μg/L exposure (**D**): widened intercellular space of the oocyte (black arrow), loss of contact between the oocyte cell membranes and the follicular cell layer (white arrow). (**E**–**H**) H.E-stained testis section. Control (**E**); 1 μg/L exposure (**F**): optically empty intercellular spaces (black arrow); 5 μg/L exposure (**G**): optically empty intercellular spaces (black arrow), cellular deteriorations (star); 20 μg/L exposure (**H**): the same lesions as described in 5 μg/L exposure.

In the ovary of control females, oocytes were connected by the gonadosomatic tissue and edged with follicular cells. Most of oocytes were filled with yolk platelets stained in red by eosin (Fig. 2A). After MC exposure, loss of contact between the oocyte cell membranes and the follicular cell layer was observed in ovary, indicating the disruption of relationships between follicular cells and oocytes. Moreover, the vacuolation and lysis of gonadosomatic tissue and widened intercellular space of oocytes were found in the treatment groups (Fig. 2B–D). In the testis of control males, cells were well shaped and cells were interrelated via cytoplasmic bridges within each tubule (Fig. 2E). In the testis from male fish exposed to MC-LR, a few cellular deteriorations and optically empty intercellular spaces were detectable under the photon microscope (Fig. 2F–H).

### Oogenesis and Spermatogenesis

**Figure 3:**
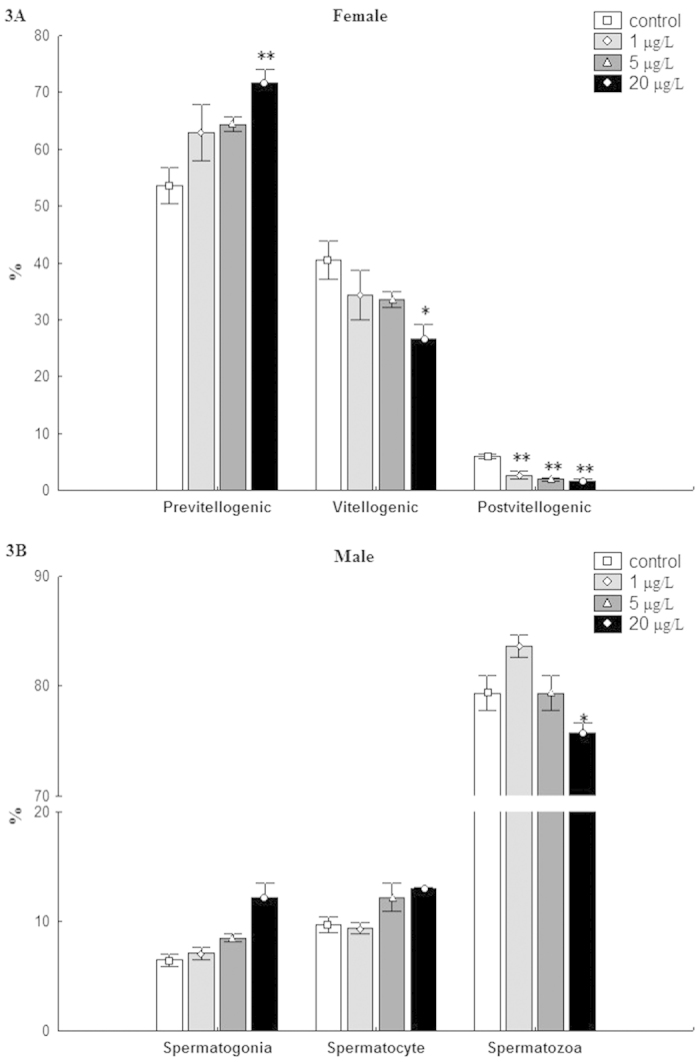
Effects of MC-LR exposure on oogenesis and spermatogenesis in zebrafish. (**A**): percentages of pre-vitellogenic, vitellogenic and post-vitellogenic oocytes in ovaries from females (mean ± S.E., n = 3) exposed to MC-LR; (**B**): percentages of spermatogonia, spermatocytes and spermatids in testes from males (mean ± S.E., n = 3) exposed to MC-LR. Asterisk (*) and (**) indicate significant differences at *P* < 0.05 and *P* < 0.01 between MC-LR treated groups and the control group, respectively.

Exposure to sublethal concentrations of MC-LR affected ovarian and spermatic development (Fig. 3). A significant increase in the number of pre-vitellogenic oocytes was observed in female fish exposed to 20 μg/L MC-LR, which was associated with significant decreases in vitellogenic and post-vitellogenic oocytes shown in the same group. When exposed to 1 μg/L or 5 μg/L MC-LR, the number of post-vitellogenic oocytes also dramatically decreased in the ovary from females. Treatment with 20 μg/L MC-LR caused an obvious decrease in the number of spermatozoa in male fish and this increase was associated with significant increases in spermatogonia and spermatocyte observed in the same group.

### Concentrations of Hormones

**Figure 4:**
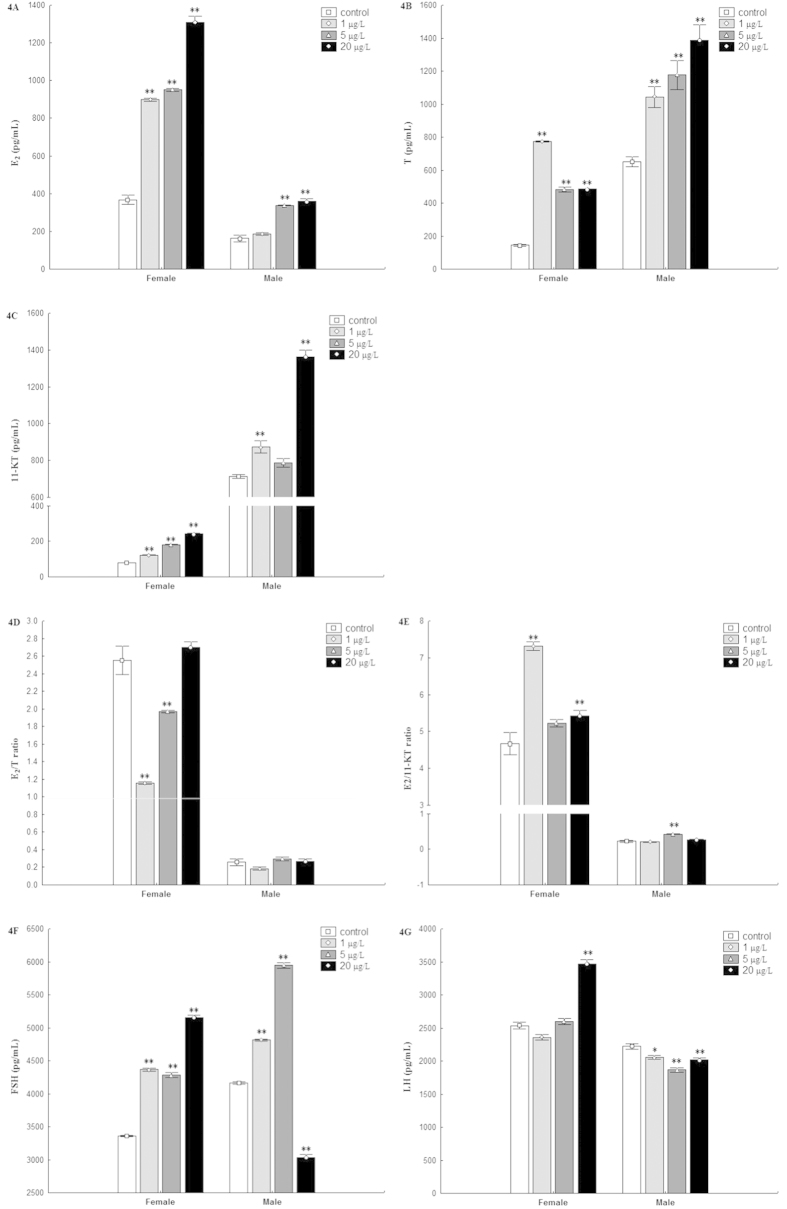
Effects of MC-LR on serum levels of (**A**) 17β-estradiol (E_2_), (**B**) testosterone (T), (**C**) 11-keto testosterone (11-KT), (**D**) E_2_/T ratio, (**E**) E2/11-KT ratio, (**F**) follicle-stimulating hormone (FSH) and (**G**) luteinizing hormone (LH) (mean ± S.E., n = 5). Asterisk (*) and (**) indicate significant differences at *P* < 0.05 and *P* < 0.01 between MC-LR treated groups and the control group, respectively.

Serum E_2_ levels increased as the exposure concentrations of MC-LR increased regardless of sex (*P* < 0.01) (Fig. 4A). There were significant changes of serum T concentrations in both females and males after exposure to MC-LR (*P* < 0.01) (Fig. 4B). Serum 11-KT concentrations increased significantly in all treatments except 5 μg/L MC-LR treated males (*P* < 0.01) (Fig. 4C). In female fish, E_2_/T ratio decreased dramatically at 1 μg/L or 5 μg/L MC-LR (*P* < 0.01) (Fig. 4D); E_2_/11-KT ratio was significantly greater in 1 μg/L or 20 μg/L MC-LR treated female fish and 5 μg/L MC-LR treated male fish (*P* < 0.01) (Fig. 4E). Serum FSH concentration increased in all treated females and 1 μg/L or 5 μg/L MC-LR treated males (*P* < 0.01); while there was a decrease in FSH levels in serum from male zerafish after exposure to 20 μg/L MC-LR (*P* < 0.01) (Fig. 4F). The concentration of LH in serum increased in female fish treated with 20 μg/L MC-LR (*P* < 0.01) and decreased in all treated males (*P* < 0.05) (Fig. 4G).

The concentrations of E_2_, T and 11-KT in female fish were negatively associated with the number of post-vitellogenic oocytes (*β* =−0.0047, *P* < 0.001; *β* = −0.0055, *P* = 0.016; *β* = −0.0232, *P* = 0.003), while serum E_2_, T and 11-KT levels in males were also negatively correlated with the number of spermatozoa (*β* = −0.0309, *P* < 0.001; *β* = −0.0059, *P* = 0.002; *β* = −0.0098, *P* = 0.018).

### Transcriptional Changes in Genes of the HPG Axis

**Figure 5:**
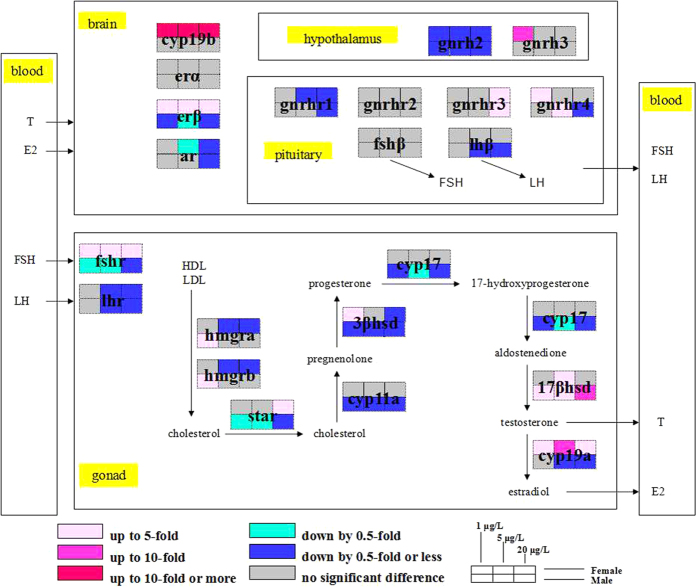
Effects of MC-LR on gene transcription in the HPG axis in male and female zebrafish. Gene transcription concentrations in female (upper) and male (lower) zebrafish treated by 1, 5 and 20 μg/L MC-LR are shown as color sets on the selected endocrine pathways along the HPG axis (n = 6). The colors describe different fold thresholds. Gene acronyms are defined in Table 1.

The transcriptions of the genes of HPG axis were affected by exposure to MC-LR (Fig. 5 and Supporting Information Table S4). In females, the mRNA levels of *cyp19b, erβ, fshr* and *cyp19a* increased in all exposed groups (*P* < 0.05), while expression of *gnrh2* were significantly decreased by exposure to MC-LR (*P* < 0.01). In males, significant down-regulation of *gnrh2, erβ, fshr, star, cyp11a, 3βhsd* and *cyp17* mRNAs were observed (*P* < 0.05) and significant up-regulation of *17βhsd* mRNAs were exhibited (*P* < 0.01) in all treatment groups.

The relationship between gene transcriptions and germ cell development in male and female fish was evaluated. PCA was applied to reduce the number of independent variables to fewer factors (PCs) based on the precondition of the high correlation of the 22 genes investigated (r > 0.5, *P* < 0.01, Supporting Information Table S5). The results of PCA showed that PC1 accounted for 49.36% of the total variances for females and 39.68% for males, while PC2 accounted for additional 17.71% for females and 18.32% for males of total variances (Supporting Information Figure S1 and Table S6). In females, major contributors to PC1 (*gnrh2, gnrhr3, ar, lhr, hmgra, hmgrb and cyp19a*) were positively correlated with the number of post-vitellogenic oocytes (*β* = 0.395, *P* = 0.005). In male zebrafish, the PC1 highly influenced by *gnrh2, lhβ, erβ, fshr, cyp11a and* 17*βhsd* had significantly correlation with the number of spermatozoa (*β* = 0.1627, *P* = 0.024).

### Western Blot Analysis

**Figure 6:**
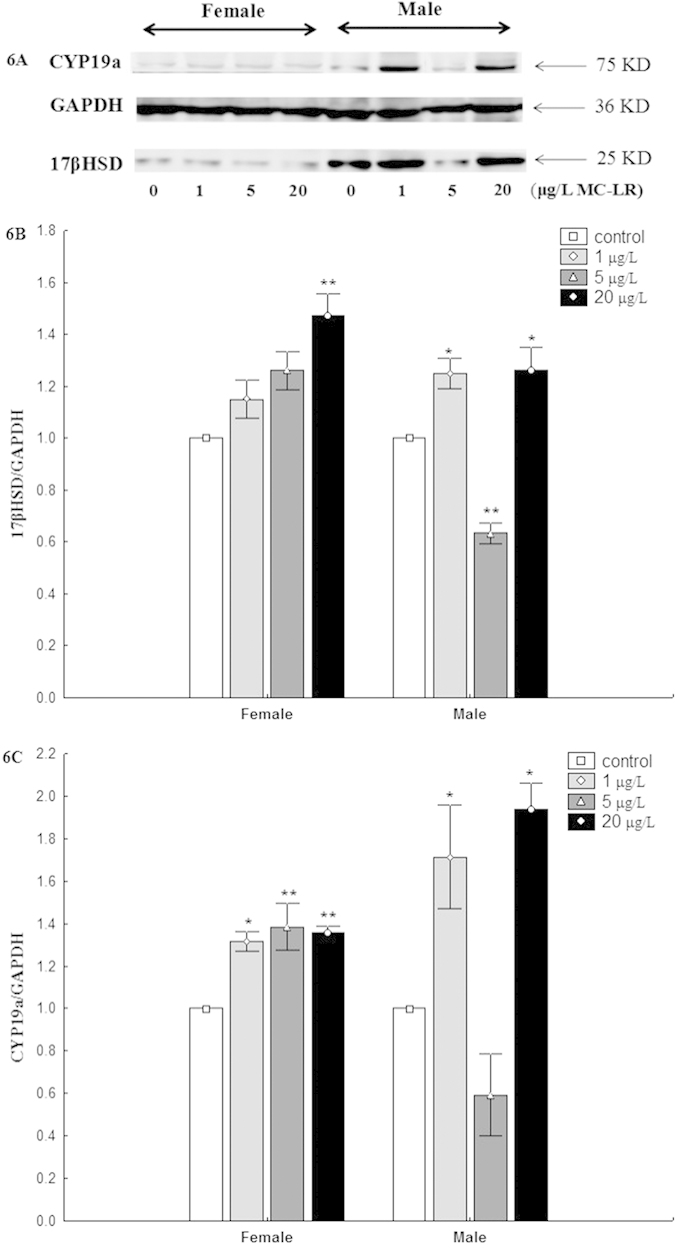
Western blot analysis of 17βHSD and CYP19a expressions in gonads of female and male zebrafish exposed to MC-LR (**A**); Bands were quantified using Quantity One 5.0 and shown as (**B**) 17βHSD/GAPDH in ovaries and testis and (**C**) CYP19a/GAPDH in ovaries and testis (mean ± S.E., n = 3). Asterisk (*) and (**) indicate significant differences at *P* < 0.05 and *P* < 0.01 between MC-LR treated groups and the control group, respectively.

The protein levels of 17βHSD and CYP19a were shown in Fig. 6. In ovary of female, the expressions of protein 17βHSD and CYP19a were increased in all treatment. In males, the expressions of protein 17βHSD and CYP19a were greater in testis after exposure to MC-LR at 1 μg/L and 20 μg/L.

## Discussion

In the present study, exposure to MC-LR in adult zebrafish resulted in sex-dependent effects on the gene networks of HPG axis, alterations of 17βHSD and CYP19a protein expressions in gonads and changes of hormone levels in serum, and eventually led to gonad damage and arrested germ cell development. Previous studies have reported that microcystins can accumulate in gonads and exert negative effects on the reproductive system^8^,^32^,^33^,^34^,^35^,^36^,^37^, and the reproductive toxicity of MCs could be gender dependent^16^,^17^. Nevertheless, the underlying sex-dependent molecular mechanism of reproductive toxicity in fish induced by MCs has not been well characterized. Therefore, we applied PCA to find some sex-specific genes in HPG axis related with gametogenesis suppression caused by MC-LR exposure and illustrated the potential mechanism of sex-dependent reproductive toxicity following MCs or other EDs exposure.

Previous studies have reported that MC-LR exposure can affect reproductive function by detecting tissue damage in gonads in medaka fish and zebrafish^13^,^16^. GSI is often used as an important indicator to evaluate the toxic effects on whole gonads^38^,^39^. Our data showed that GSI was dramatically decreased in males in all treatments and female fish exposed to 20 μg/L MC-LR. Furthermore, various histopathological alterations were observed in the gonads of fish treated with MC-LR in the present study, including cellular deteriorations and empty intercellular spaces in testis, vacuolation and lysis of gonadosomatic tissue and widened intercellular space of oocytes in ovary. These histopathological changes were shared by our previous study in zebrafish treated with MC-LR^16^.

Our previous study showed that MC-LR exposure to parental zebrafish evidently influenced the growth in F1 offspring^30^. We considered this damage as a parental transmission effect of microcystin toxicity, which indicated a defect in oogenesis and spermatogenesis in adult zebrafish. Zhao *et al*. reported that MC-LR impaired zebrafish reproduction by affecting oogenesis^20^. However, the potential molecular mechanism of the disruption of gametogenesis in fish induced by MC-LR is still relatively insufficiently known.

Compared to the control group, the development of germ cell was influenced by MC-LR treatment in both male and female zebrafish. The present results showed that MC-LR exposure caused a marked reduction in the proportion of spermatozoa in 20 μg/L treated male fish, which might contribute to the decreased fertilization success following the treatment. In the present study, a significant increase in pre-vitellogenic stages and decreases in vitellogenic and post-vitellogenic stages were also observed in females at ≥1 μg/L MC-LR, indicating that MC-LR exposure might lead to follicular developmental stages shifting towards the immature stages. Other potential EDs, such as bis-(2-ethylhexyl)-phthalate (DEHP), also induced defects in oogenesis and spermatogenesis in zebrafish^31^,^40^. We suggest that gametogenesis should be critical parameters when evaluating endocrine disruption and reproductive toxicology caused by environmental compounds.

The results of PCA showed the relationship between gene transcriptions and gametogenesis, and the cluster of the genes constituting the first two PCs were different between male and female fish. In female zebrafish, PC1 (*gnrh2, gnrhr3, ar, lhr, hmgra, hmgrb and cyp19a*) were positively correlated with the number of post-vitellogenic oocytes while major contributors to PC1 (*gnrh2, lhβ, erβ, fshr, cyp11a and* 17*βhsd*) in males were also positively correlated with the number of spermatozoa. It was worth mentioning that the major genes influencing gametogenesis were different between females and males, suggesting adverse effects of MC-LR on germ cell development and gene transcriptions were sex dependent. We speculate that these specific gene transcriptions might be used as biomarkers to explore the gender-dependent effects of MCs or other EDs.

In male fish, FSH and LH are the most important pituitary hormones regulating spermatogenesis^41^. FSH plays a regulatory role during early stages of spermatogenesis, and LH is mainly involved in later stages of maturation, e.g., regulating spermiation^41^,^42^. In this study, transcriptions of *lhβ* and *lhr* genes in males decreased after exposure to MC-LR at 5 μg/L and 20 μg/L groups, which could inhibit the secretion of LH from pituitary and subsequent transport to gonad and was consistent with the lesser concentrations of LH in serum from all MC-LR treated zebrafish. It was noteworthy that MC-LR reduced the proportion of spermatozoa dramatically in male zebrafish at 20 μg/L treatment, which was consistent with the lesser transcriptions of *lhβ, lhr* and *fshr* genes in testis and the declines of LH and FSH in serum. Some studies have demonstrated that spermatogonial cells could be the target of MC-LR^43^,^44^,^45^. Zhou *et al*. reported that MC-LR modulated the apoptosis and proliferation of spermatogonia and altered the expression of specific genes *in vivo*^45^. We speculate that MC-LR exposure might inhibit the translation of spermatogonia to spermatozoa in males by disrupting the mRNA expression of genes and pituitary hormones on HPG axis. During the process of producing sex steroid hormones in the testis, down-regulation of *star, cyp11a, 3βhsd* and *cyp17* had the potential to decrease testosterone in males. However, the alterations of these genes mRNA levels detected in all treatments did not decrease the serum levels of testosterone in male zebrafish. In HPG axis, the increase in *17βhsd* mRNAs and decrease in *cyp19a* mRNAs agreed with the greater levels of T and 11-KT. The increases of T and 11-KT might be a consequence of testis damage induced by MC-LR. Furthermore, the protein levels of 17βHSD amd CYP19a increased in testis from treated males at 20 μg/L group, which was in accordance with the greater productions of sex hormones (T, 11-KT and E_2_) in serum. The lesser transcriptions of *erβ* in males might be a response to the greater concentration of E_2_ as a negtive feedback^28^, which could regulate the synthesis of sex hormones in gonad by mRNA levels of genes in brain.

Lesser production of *gnrh2* and *gnrhr1* were observed in brain from female zebrafish exposed to MC-LR. In vertebrates, GnRH has a crucial role on the control of reproduction through HPG axis. GnRH can regulate the synthesis and release of gonadotropin hormone (LH and FSH) closely related with oogenesis^27^. We deduce that the down-regulation of *gnrhr1* and *gnrhr4* in brain and decreases in *lhr* mRNA levels in ovary might inhibit the transport of LH from pituitary to target organism gonad, consistent with the suppression of oogenesis in female fish. In addition, lesser transcriptions of *erβ*, possible consequence of broken negative E_2_ feedback loop induced by MC-LR, were in accordance with the significant increases of E_2_ and T in females exposed to MC-LR. In fish brain, *cyp19b* is known to be controlled by a positive auto-regulatory feedback loop driven by E_2_^46^. Greater production of *cyp19b* in females treated with MC-LR mighe be a response to a greater level of E_2_^28^. The up-regulation of *cyp19a* might be a response to arrested oogenesis. The defects in oogenesis caused by EDs including MC-LR might be attributable to a direct effect on ovary or to an indirect disruption of the normal HPG endocrine system.

Sex steroid hormones (E_2_ and T or 11-KT) play crucial roles in various parameters associated with reproduction in fish, such as fecundity rate, fertilization rate, hatch success and survival of embryos^47^. So measurement of sex steroid hormones has been suggested to be one of the most integrative and functional endpoints for understanding the effects of an ED on reproduction in zebrafish^48^,^49^. In the present study, serum E_2_, T and 11-KT levels increased in all MC-LR exposed zebrafish regardless of sex. The results indicated the alterations of sex hormone balance induced by MC-LR^20^,^50^. We also found that number of eggs spawned and hatchability decreased in the MC-LR treated groups^16^, which was consistent with defects of spermatic and ovarian development. Some studies have proved alterations of sex hormone levels may cause reproductive dysfunction by disrupting the regulatory mechanisms of HPG axis^27^,^28^. Our results demonstrated that MC-LR would affect the success of reproduction in zebrafish by its endocrine disrupting influences on the HPG axis. We deduce that MC-LR can result in substantial toxicity to zebrafish reproduction, causing decrease in GSI, injury to gonad structure and suppression in gametogenesis by changing the transcripts of genes in HPG axis and disrupting the balance of sex hormones.

In the present study, MC-LR altered the mRNA levels of genes along the HPG axis (Supporting Information Figure S2); especially in the 20 μg/L MC group, down-regulation of 7 genes (*P* < 0.05) and up-regulation of 6 genes (*P* < 0.05) were detected in female fish, and down-regulation of 13 genes (*P* < 0.05) and down-regulation of 2 genes (*P* < 0.01) were observed in male fish. The results demonstrated that balance among the gene transcriptions was disrupted by MC-LR and MC exposure tended to decrease the gene mRNA levels in male fish. Some other studies also reported that MC-LR could damage the HPG system^20^,^51^,^52^.

In summary, our results clearly showed that exposure to MC-LR could disrupt the function of HPG endocrine system in fish, including alterations of sex hormone levels, protein levels and gene transcriptions accompanied by decreasing the GSI, impairing the gonads and disrupting the gametogenesis in zebrafish. Furthermore, the responses to MC-LR in zebrafish were sex dependent. To the best of our knowledge, this study is the first report which links the transcriptions of genes of HPG axis to gametogenesis in both females and males exposed to MC-LR and evaluates the sex-specific effects endocrine disruption and reproductive toxicity caused by MC-LR.

## Additional Information

**How to cite this article**: Liu, W. *et al*. Sex-dependent effects of microcystin-LR on hypothalamic-pituitary-gonad axis and gametogenesis of adult zebrafish. *Sci. Rep.* **6**, 22819; doi: 10.1038/srep22819 (2016).

## Supplementary Material

Supplementary Information

## Figures and Tables

**Table 1:** Gene list of HPG axis of zebrafish.

Abbreviation	Gene name	Category
*gnrh*	Gonadotropin-releasing hormone	Hormone
*gnrhr*	Gonadotropin-releasing hormone receptor	Peptide receptor
*fshβ*	Follicle stimulating hormone β	Hormone
*lhβ*	Luteinizing hormone β	Hormone
*cyp19b*	Cytochrome P450 19B	Steroidogenesis
*er*	Estrogen receptor	Steroidogenesis
*ar*	Androgen receptor	Steroidogenesis
*fshr*	Follicle stimulating hormone receptor	Peptide receptor
*lhr*	Luteinizing hormone receptor	Peptide receptor
*hmgr*	Hydroxymethylglutaryl CoA reductase	Steroidogenesis
*star*	Steroidogenic acute regulatory protein	Steroidogenesis
*cyp11a*	Cytochrome P450 side-chain cleavage	Steroidogenesis
*3βhsd*	3β-hydroxysteroid dehydrogenase	Steroidogenesis
*cyp17*	Cytochrome P450 17	Steroidogenesis
*17βhsd*	17β-hydroxysteroid dehydrogenase	Steroidogenesis
*cyp19a*	Cytochrome P450 19A	Steroidogenesis
