# Supplementary material for: Sex-dependent effects of microcystin-LR on hypothalamic-pituitary-gonad axis and gametogenesis of adult zebrafish
Source: Sci Rep. 2016 Mar 10;6:22819. doi: 10.1038/srep22819 (PMC4785373; doi:10.1038/srep22819)
Supplement: Supplementary Information [file srep22819-s1.doc]

**SUPPORTING INFORMATION**

Sex-dependent effects of microcystin-LR on hypothalamic-pituitary-gonad axis and gametogenesis of adult zebrafish

Wanjing Liu^a^, Chuanyue Chen^a^, Liang Chen^b^, Li Wang^a^, Jian Li^a^, Yuanyuan Chen^a^, Jienan Jin^a^, Atufa Kawan^a^, Xuezhen Zhang*^, a^

^a^ College of Fisheries, Huazhong Agricultural University, Freshwater Aquaculture Collaborative Innovation Center of Hubei Province, Institute of Hydrobiology, Chinese Academy of Sciences, Wuhan 430070, People’s Republic of China

^b^ Donghu Experimental Station of Lake Ecosystems, State Key Laboratory of Freshwater Ecology and Biotechnology, Institute of Hydrobiology, Chinese Academy of Sciences, Wuhan 430072, People’s Republic of China

^*^Corresponding author:

Xuezhen Zhang, Ph.D.

College of Fisheries, Huazhong Agricultural University, Shizishan street 1, Wuhan

430070, P.R. China.

Phone and fax: +86-27-87282114

E-mail: xuezhen@mail.hzau.edu.cn


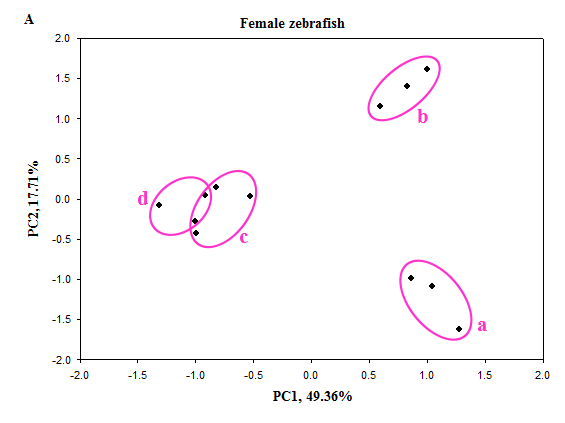


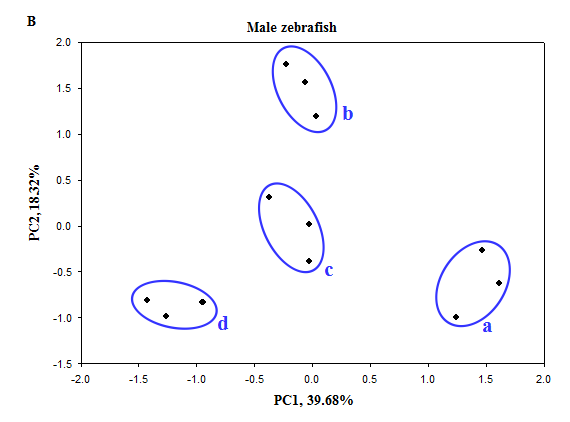


**Figure S1.** Plot of first two factors of principal component analysis of gene transcriptions along

the HPG axis. Clusters a-d represent control, 1 μg/L, 5 μg/L and 20 μg/L MC-LR group, respectively. (A) Female, (B) Male.

.


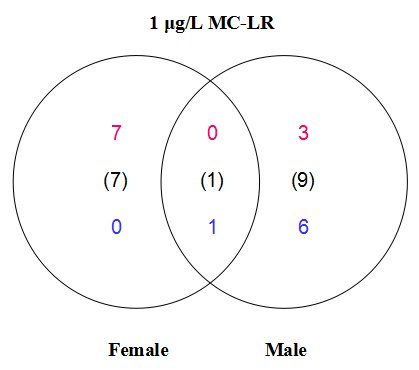

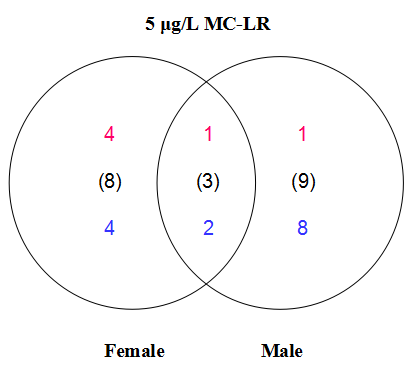


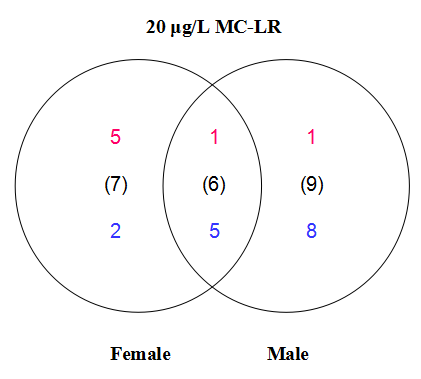

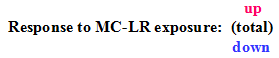


**Figure S2.** Venn diagram showing the number of differentially expressed genes of the HPG axis detected in the present study. Black numbers in parentheses represent totals, and red numbers and blue numbers reprensent up-regulted genes and down-regulated genes, respectively.

Table S1. Concentration (μg/L) of MC-LR measured in exposure water during the 30 d assay.

| Treatment (nominal concentration ) | Measured concentration of MC-LR^a^ | | | | | | |
| --- | --- | --- | --- | --- | --- | --- | --- |
|  | Days of exposure | | | | | | |
|  | 1 | 6 | 12 | 18 | 24 | 30 | Mean |
| Control (0) | <MDL^b^ | <MDL | <MDL | <MDL | <MDL | <MDL | - |
| 1 | 0.85  (0.01) | 0.93 (0.03) | 0.90  (0.03) | 0.86  (0.02) | 0.84  (0.01) | 0.88  (0.02) | 0.88  (0.01) |
| 5 | 4.63  (0.12) | 4.69  (0.07) | 4.65  (0.08) | 4.51  (0.04) | 4.75  (0.07) | 4.47  (0.08) | 4.62  (0.04) |
| 20 | 18.37  (0.25) | 19.04  (0.39) | 18.81  (0.41) | 18.61  (0.18) | 18.44  (0.29) | 18.33  (0.19) | 18.60  (0.11) |

^a^ Values are means (standard error) of 3 replicates of each treatment.

^b^ Minimum detection limit (MDL) is 0.1 μg/L.

**Table S2.** Quantitative polymerase chain reaction (qPCR) primers used for gene expression assays.

| Gene name | Accession No. | Description | Sequence (5’-3’) |
| --- | --- | --- | --- |
| *β-actin* | NM_131031 | Forward | TGCTGTTTTCCCCTCCATTG |
|  |  | Reverse | TCCCATGCCAACCATCACT |
| *gnrh2* | AY657018 | Forward | CTGAGACCGCAGGGAAGAAA |
|  |  | Reverse | TCACGAATGAGGGCATCCA |
| *gnrh3* | NM_182887 | Forward | TTGCCAGCACTGGTCATACG |
|  |  | Reverse | TCCATTTCACCAACGCTTCTT |
| *gnrhr1* | NM_001144980 | Forward | ACCCGAATCCTCGTGGAAA |
|  |  | Reverse | TCCACCCTTGCCCTTACCA |
| *gnrhr2* | NM_001144979 | Forward | CAACCTGGCCGTGCTTTACT |
|  |  | Reverse | GGACGTGGGAGCGTTTTCT |
| *gnrhr3* | NM_001177450 | Forward  Reverse | GAGGCGCAGCGGAACA  GTCATCTTCAGCGTCTTCATCCT |
| *gnrhr4* | NM_001098193 | Forward | CACCAACAACAAGCGCAAGT |
|  |  | Reverse | GGCAACGGTGAGGTTCATG |
| *fshβ* | NM_205624 | Forward | GCTGTCGACTCACCAACATCTC |
|  |  | Reverse | GTGACGCAGCTCCCACATT |
| *lhβ* | NM_205622 | Forward | GGCTGCTCAGAGCTTGGTTT |
|  |  | Reverse | TCCACCGATACCGTCTCATTTA |
| *cyp19b* | AF183908 | Forward | GTCGTTACTTCCAGCCATTCG |
|  |  | Reverse | GCAATGTGCTTCCCAACACA |
| *erα* | NM_152959 | Forward | CAGACTGCGCAAGTGTTATGAAG |
|  |  | Reverse | CGCCCTCCGCGATCTT |
| *erβ* | NM_174862 | Forward | TTCACCCCTGACCTCAAGCT |
|  |  | Reverse | TCCATGATGCCTTCAACACAA |
| *ar* | NM_001083123 | Forward | TCTGGGTTGGAGGTCCTACAA |
|  |  | Reverse | GGTCTGGAGCGAAGTACAGCAT |
| *fshr* | NM_001001812 | Forward | CGTAATCCCGCTTTTGTTCCT |
|  |  | Reverse | CCATGCGCTTGGCGATA |
| *lhr* | AY424302 | Forward | AAAAGGACGAGTCGCTGAAA |
|  |  | Reverse | GCTCTTCTGGGAACATCTGC |
| *cyp17* | AY281362 | Forward | TCTTTGACCCAGGACGCTTT |
|  |  | Reverse | CCGACGGGCAGCACAA |
| *cyp19a* | AF226620 | Forward | GCTGACGGATGCTCAAGGA |
|  |  | Reverse | CCACGATGCACCGCAGTA |
| *cyp11a* | NM_152983 | Forward | GGCAGAGCACCGCAAAA |
|  |  | Reverse | CCATCGTCCAGGGATCTTATTG |
| *star* | NM_131663 | Forward  Reverse | GGTCTGAGGAAGAATGCAATGAT  CCAGGTCCGGAGAGCTTGT |
| *hmgra* | BC155135 | Forward  Reverse | GAGCCATCGACTCTCTCCTG  GAACACGACTGCTAGCACCA |
| *hmgrb* | NM_001014292 | Forward  Reverse | CCAACTGCTCCCTGATCAAT  TTTAAGAATCGCGAGGCACT |
| *3βhsd* | AY279108 | Forward | AGAGACCCGGAGAAAAG |
|  |  | Reverse | GGGTGGAGTGAAATCTCAGGA |
| *17βhsd* | AY306005 | Forward | GTCTGATGGGTCCTCT |
|  |  | Reverse | TCTCACAAGCGCCCTCTATT |

**Table S3.** Information of the antibodies.Gene list of HPG axis of zebrafish.

|  | Anti-17βHSD antibody | Anti-CYP19a antibody |
| --- | --- | --- |
| Host | Goat | Rabbit |
| Clonality | Polyclonal | Polyclonal |
| Species Reactivity | Zebrafish, Huma,n, Mouse, Rat | Zebrafish |
| Applications | WB, IHC | WB |

**Table S4.** Transcriptional response profiles of genes in HPG axis in female and male zebrafish

after the exposure to MC-LR.^a^

| **Tissue** | **Gene** | **MC-LR / Female** | | | **MC-LR / Male** | | |
| --- | --- | --- | --- | --- | --- | --- | --- |
|  |  | **1 μg/L** | **5 μg/L** | **20 μg/L** | **1 μg/L** | **5 μg/L** | **20 μg/L** |
| **Brain** | gnrh2 | 0.31 ± 0.09^**^ | 0.04 ± 0.01^**^ | 0.03 ± 0.01^**^ | 0.42 ± 0.12^**^ | 0.41 ± 0.12^**^ | 0.18 ± 0.03^**^ |
|  | gnrh3 | 5.57 ± 0.97^**^ | 1.42 ± 0.42 | 2.16 ± 0.41 | 0.88 ± 0.14 | 0.76 ± 0.19 | 0.67 ± 0.18 |
|  | gnrhr1 | 0.55 ± 0.18 | 0.18 ± 0.09^*^ | 0.15 ± 0.05^*^ | 1.38 ± 0.31 | 1.57 ± 0.22 | 0.11 ± 0.06^**^ |
|  | gnrhr2 | 0.67 ± 0.14 | 0.56 ± 0.10 | 0.85 ± 0.23 | 0.91 ± 0.12 | 0.77 ± 0.13 | 0.61 ± 0.20 |
|  | gnrhr3 | 1.34 ± 0.22 | 2.17 ± 0.08 | 2.46 ± 0.34^*^ | 2.65 ± 0.45 | 1.41 ± 0.52 | 3.92 ± 0.84^**^ |
|  | gnrhr4 | 1.99 ± 0.27^*^ | 0.62 ± 0.18 | 0.61 ± 0.16 | 0.78 ± 0.10 | 2.27 ± 0.21^**^ | 0.42 ± 0.07^*^ |
|  | fshβ | 1.00 ± 0.42 | 1.04 ± 0.15 | 1.66 ± 0.29 | 0.75 ± 0.10 | 1.10 ± 0.22 | 1.14 ± 0.09 |
|  | lhβ | 1.13± 0.34 | 0.65 ± 0.15 | 0.30 ± 0.15 | 0.51 ± 0.06 | 0.45 ± 0.14^*^ | 0.42 ± 0.10^*^ |
|  | cyp19b | 10.52 ± 1.33^**^ | 12.10 ± 1.25^**^ | 16.74 ± 1.06^**^ | 0.91 ± 0.10 | 1,31 ± 0.20 | 0.85 ± 0.17 |
|  | erα | 0.71 ± 0.08 | 1.5 ± 0.26 | 1.72 ± 0.23 | 0.89 ± 0.14 | 1.12 ± 0.02 | 0.06 ± 0.26 |
|  | erβ | 4.95 ± 0.55^**^ | 3.04 ± 0.40^*^ | 3.08 ± 0.45^*^ | 0.31 ± 0.19^**^ | 0.59 ± 0.13^*^ | 0.19 ± 0.03^**^ |
|  | ar | 1.02± 0.13 | 0.51 ± 0.14^*^ | 0.22 ± 0.04^**^ | 0.86 ± 0.07 | 0.91 ± 0.11 | 0.40 ± 0.12^**^ |
| **Gonad** | fshr | 2.39 ± 0.13^**^ | 2.65 ± 0.40^**^ | 3.07 ± 0.32^**^ | 0.71 ± 0.02^**^ | 0.55 ± 0.05^**^ | 0.37 ± 0.03^**^ |
|  | lhr | 0.83 ± 0.16 | 0.35 ± 0.09^**^ | 0.18 ± 0.07^**^ | 0.74 ± 0.14 | 0.47 ± 0.13^*^ | 0.30 ± 0.09^**^ |
|  | hmgra | 1.15 ± 0.09 | 0.31 ± 0.17^**^ | 0.21 ± 0.04^**^ | 4.24 ± 0.31^**^ | 2.00 ± 0.44 | 2.02 ± 0.32 |
|  | hmgrb | 0.63 ± 0.11 | 0.28 ± 0.10^**^ | 0.06 ± 0.01^**^ | 2.72 ± 0.29^**^ | 0.65 ± 0.07 | 0.52 ± 0.13 |
|  | star | 1.59 ± 0.30 | 2.04 ± 0.31 | 3.88 ± 0.82^**^ | 0.57 ± 0.11^**^ | 0.52 ± 0.06^**^ | 0.44 ± 0.06^**^ |
|  | cyp11a | 1.02 ± 0.09 | 1.37 ± 0.14 | 1.60 ± 0.21 | 0.37 ± 0.06^**^ | 0.36 ± 0.06^**^ | 0.20 ± 0.07^**^ |
|  | cyp17 | 0.89 ± 0.06 | 1.28 ± 0.22 | 1.24 ± 0.11 | 0.26 ± 0.05^**^ | 0.57 ± 0.03^*^ | 0.45 ± 0.09^**^ |
|  | 3βhsd | 1.59 ± 0.15^*^ | 0.66 ± 0.09 | 0.44 ± 0.06^**^ | 0.29 ± 0.09^**^ | 0.28 ± 0.12^**^ | 0.31 ± 0.19 ^*^ |
|  | 17βhsd | 0.71 ± 0.02 | 1.94 ± 0.12^**^ | 1.21 ± 0.12 | 4.21 ± 0.17^**^ | 2.28 ± 0.21^**^ | 7.79 ± 0.11^**^ |
|  | cyp19a | 2.26 ± 0.16^*^ | 6.04 ± 0.70^**^ | 4.32 ± 0.64^**^ | 1.09 ± 0.10 | 0.43 ± 0.12^*^ | 0.42 ± 0.09^*^ |

^a^ mRNA expression is expressed as the fold change compared to the corresponding control mRNA expression (mean ± S.E., n = 6). Asterisk (*) and (**) indicate significant differences at *P* < 0.05 and *P* < 0.01 between MC-LR treated groups and the control group, respectively.

.

**Table S5.** Spearman correlation coefficients (r) between mRNA expressions of the genes along the HPG axis in male and female zebrafish after MC-LR exposure

| **Female** |  | **Brain** | | | | | | | | | | | |  | **Gonad** | | | | | | | | | |
| --- | --- | --- | --- | --- | --- | --- | --- | --- | --- | --- | --- | --- | --- | --- | --- | --- | --- | --- | --- | --- | --- | --- | --- | --- |
|  |  | gnrh2 | gnrh3 | gnrhr1 | gnrhr2 | gnrhr3 | gnrhr4 | fshβ | lhβ | cyp19b | erα | erβ | ar |  | fshr | lhr | hmgra | hmgrb | star | cyp11a | 3βhsd | cyp17 | 17βhsd | cyp19a |
| **Brain** |  |  |  |  |  |  |  |  |  |  |  |  |  |  |  |  |  |  |  |  |  |  |  |  |
| ghrh2 |  | 1.000 | 0.112 | **0.782** | 0.287 | **-0.780** | 0.632 | -0.270 | 0.518 | **-0.804** | **-0.559** | -0.048 | 0.481 |  | **-0.689** | **0.633** | **0.702** | **0.741** | **-0.638** | -0.374 | **0.631** | -0.417 | -0.515 | **-0.874** |
|  |  |  | (0.679) | **(0.002)** | (0.282) | **(0.003)** | (0.11) | (0.372) | (0.070) | **(0.001)** | **(0.020)** | (0.864) | (0.059) |  | **(0.004)** | **(0.006)** | **(0.001)** | **(0.000)** | **(0.006)** | (0.126) | **(0.009)** | (0.067) | (0.072) | **(0.000)** |
| gnrh3 |  |  | 1.000 | 0.105 | -0.160 | -0.193 | 0.468 | -0.116 | 0.099 | 0.200 | -0.389 | 0.546 | 0.365 |  | 0.191 | 0.057 | 0.252 | 0.041 | 0.186 | -0.239 | 0.479 | -0.403 | -0.573 | -0.044 |
|  |  |  |  | (0.746) | (0.584) | (0.549) | (0.079) | (0.721) | (0.748) | (0.493) | (0.152) | (0.035) | (0.181) |  | (0.512) | (0.839) | (0.347) | (0.879) | (0.508) | (0.390) | (0.071) | (0.121) | (0.051) | (0.887) |
| gnrhr1 |  |  |  | 1.000 | 0.346 | **-0.897** | 0.392 | -0.079 | 0.643 | -0.622 | -0.522 | -0.280 | **0.836** |  | -0.680 | **0.722** | 0.637 | **0.785** | -0.676 | -0.527 | 0.636 | -0.245 | -0.516 | -0.748 |
|  |  |  |  |  | (0.247) | **(0.000)** | (0.208) | (0.503) | (0.024) | (0.031) | (0.067) | (0.379) | **(0.000)** |  | (0.011) | **(0.008)** | (0.019) | **(0.001)** | (0.011) | (0.064) | (0.026) | (0.420) | (0.071) | (0.1005) |
| gnrhr2 |  |  |  |  | 1.000 | 0.137 | -0.455 | 0.157 | 0.007 | 0.022 | 0.153 | -0.401 | 0.208 |  | -0.099 | 0.011 | 0.281 | 0.231 | 0.097 | 0.055 | -0.288 | -0.234 | 0.033 | -0.484 |
|  |  |  |  |  |  | (0.672) | (0.102) | (0.609) | (0.983) | (0.943) | (0.571) | (0.174) | (0.458) |  | (0.736) | (0.971) | (0.292) | (0.389) | (0.721) | (0.852) | (0.318) | (0.383) | (0.915) | (0.094) |
| gnrhr3 |  |  |  |  |  | 1.000 | -0.452 | 0.309 | -0.648 | 0.676 | 0.708 | 0.221 | **-0.860** |  | 0.570 | **-0.726** | -0.704 | **-0.777** | 0.704 | 0.420 | **-0.729** | 0.204 | 0.578 | **0.764** |
|  |  |  |  |  |  |  | (0.140) | (0.329) | (0.023) | (0.016) | (0.010) | (0.491) | **(0.000)** |  | (0.053) | **(0.007)** | (0.011) | **(0.003)** | (0.011) | (0.174) | **(0.007)** | (0.526) | (0.049) | **(0.004)** |
| gnrhr4 |  |  |  |  |  |  | 1.000 | -0.361 | 0.401 | -0.525 | -0.604 | 0.345 | 0.357 |  | -0.436 | 0.545 | 0.504 | 0.512 | -0.538 | -0.477 | **0.788** | -0.256 | -0.678 | -0.385 |
|  |  |  |  |  |  |  |  | (0.249) | (0.174) | (0.054) | (0.022) | (0.227) | (0.020) |  | (0.119) | (0.044) | (0.055) | (0.051) | (0.047) | (0.085) | **(0.000)** | (0.358) | (0.015) | (0.194) |
| fshβ |  |  |  |  |  |  |  | 1.000 | -0.004 | 0.021 | 0.314 | -0.077 | -0.470 |  | 0.532 | -0.437 | -0.094 | -0.352 | 0.418 | 0.314 | -0.410 | 0.208 | 0.237 | -0.081 |
|  |  |  |  |  |  |  |  |  | (0.991) | (0.948) | (0.297) | (0.812) | (0.105) |  | (0.061) | (0.156) | (0.761) | (0.293) | (0.155) | (0.297) | (0.168) | (0.495) | (0.436) | (0.803) |
| lhβ |  |  |  |  |  |  |  |  | 1.000 | **-0.720** | -0.678 | 0.104 | **0.760** |  | -0.300 | 0.525 | 0.389 | 0.603 | -0.629 | -0.148 | 0.671 | 0.182 | -0.371 | -0.615 |
|  |  |  |  |  |  |  |  |  |  | **(0.006)** | (0.015) | (0.734) | **(0.004)** |  | (0.320) | (0.065) | (0.194) | (0.029) | (0.028) | (0.629) | (0.012) | (0.553) | (0.236) | (0.033) |
| cyp19b |  |  |  |  |  |  |  |  |  | 1.000 | 0.566 | 0.248 | -0.468 |  | 0.680 | **-0.687** | -0.488 | **-0.789** | **0.692** | 0.323 | -0.603 | -0.103 | 0.217 | 0.699 |
|  |  |  |  |  |  |  |  |  |  |  | (0.044) | (0.392) | (0.107) |  | (0.011) | **(0.007)** | (0.076) | **(0.001)** | **(0.009)** | (0.260) | (0.022) | (0.725) | (0.499) | (0.011) |
| erα |  |  |  |  |  |  |  |  |  |  | 1.000 | -0.222 | -0.502 |  | 0.242 | -0.453 | -0.390 | -0.399 | 0.412 | 0.357 | -0.543 | 0.135 | 0.456 | 0.346 |
|  |  |  |  |  |  |  |  |  |  |  |  | (0.446) | (0.047) |  | (0.404) | (0.104) | (0.122) | (0.112) | (0.100) | (0.191) | (0.045) | (0.606) | (0.117) | (0.247) |
| erβ |  |  |  |  |  |  |  |  |  |  |  | 1.000 | -0.033 |  | 0.542 | -0.129 | -0.173 | -0.283 | 0.169 | -0.089 | 0.167 | -0.191 | -0.294 | 0.217 |
|  |  |  |  |  |  |  |  |  |  |  |  |  | (0.911) |  | (0.056) | (0.647) | (0.537) | (0.307) | (0.563) | (0.752) | (0.568) | (0.495) | (0.354) | (0.499) |
| ar |  |  |  |  |  |  |  |  |  |  |  |  | 1.000 |  | -0.478 | 0.553 | **0.617** | **0.680** | -0.542 | -0.460 | **0.782** | -0.462 | **-0.702** | **-0.713** |
|  |  |  |  |  |  |  |  |  |  |  |  |  |  |  | (0.084) | (0.040) | **(0.008)** | **(0.003)** | (0.030) | (0.085) | **(0.001)** | (0.062) | **(0.008)** | **(0.006)** |
| **Gonad** |  |  |  |  |  |  |  |  |  |  |  |  |  |  |  |  |  |  |  |  |  |  |  |  |
| fshr |  |  |  |  |  |  |  |  |  |  |  |  |  |  | 1.000 | -0.598 | -0.459 | **-0.706** | **0.682** | 0.224 | -0.454 | 0.268 | 0.267 | 0.520 |
|  |  |  |  |  |  |  |  |  |  |  |  |  |  |  |  | (0.031) | (0.085) | **(0.003)** | **(0.007)** | (0.441) | (0.103) | (0.334) | (0.378) | (0.069) |
| lhr |  |  |  |  |  |  |  |  |  |  |  |  |  |  |  | 1.000 | **0.633** | **0.880** | -0.545 | -0.510 | **0.731** | 0.009 | -0.333 | -0.694 |
|  |  |  |  |  |  |  |  |  |  |  |  |  |  |  |  |  | **(0.006)** | **(0.000)** | (0.044) | (0.036) | **(0.001)** | (0.974) | (0.291) | (0.012) |
| hmgra |  |  |  |  |  |  |  |  |  |  |  |  |  |  |  |  | 1.000 | **0.782** | -0.598 | -0.608 | **0.639** | **-0.629** | **-0.764** | **-0.830** |
|  |  |  |  |  |  |  |  |  |  |  |  |  |  |  |  |  |  | **(0.000)** | (0.011) | (0.010) | **(0.006)** | **(0.002)** | **(0.002)** | **(0.000)** |
| hmgrb |  |  |  |  |  |  |  |  |  |  |  |  |  |  |  |  |  | 1.000 | **-0.697** | -0.542 | **0.772** | -0.306 | -0.444 | **-0882** |
|  |  |  |  |  |  |  |  |  |  |  |  |  |  |  |  |  |  |  | **(0.002)** | (0.020) | **(0.000)** | (0.177) | (0.129) | **(0.000)** |
| star |  |  |  |  |  |  |  |  |  |  |  |  |  |  |  |  |  |  | 1.000 | 0.350 | **-0.697** | 0.336 | 0.582 | 0.588 |
|  |  |  |  |  |  |  |  |  |  |  |  |  |  |  |  |  |  |  |  | (0.201) | **(0.006)** | (0.187) | (0.037) | (0.035) |
| cyp11a |  |  |  |  |  |  |  |  |  |  |  |  |  |  |  |  |  |  |  | 1.000 | -0.422 | 0.269 | 0.407 | 0.357 |
|  |  |  |  |  |  |  |  |  |  |  |  |  |  |  |  |  |  |  |  |  | (0.117) | (0.297) | (0.168) | (0.255) |
| 3βhsd |  |  |  |  |  |  |  |  |  |  |  |  |  |  |  |  |  |  |  |  | 1.000 | -0.309 | -0.657 | -0.555 |
|  |  |  |  |  |  |  |  |  |  |  |  |  |  |  |  |  |  |  |  |  |  | (0.245) | (0.020) | (0.049) |
| cyp17 |  |  |  |  |  |  |  |  |  |  |  |  |  |  |  |  |  |  |  |  |  | 1.000 | 0.641 | 0.308 |
|  |  |  |  |  |  |  |  |  |  |  |  |  |  |  |  |  |  |  |  |  |  |  | (0.018) | (0.306) |
| 17βhsd |  |  |  |  |  |  |  |  |  |  |  |  |  |  |  |  |  |  |  |  |  |  | 1.000 | 0.601 |
|  |  |  |  |  |  |  |  |  |  |  |  |  |  |  |  |  |  |  |  |  |  |  |  | (0.039) |
| cyp19a |  |  |  |  |  |  |  |  |  |  |  |  |  |  |  |  |  |  |  |  |  |  |  | 1.000 |

| **Male** |  | **Brain** | | | | | | | | | | | |  | **Gonad** | | | | | | | | | |
| --- | --- | --- | --- | --- | --- | --- | --- | --- | --- | --- | --- | --- | --- | --- | --- | --- | --- | --- | --- | --- | --- | --- | --- | --- |
|  |  | gnrh2 | gnrh3 | gnrhr1 | gnrhr2 | gnrhr3 | gnrhr4 | fshβ | lhβ | cyp19b | erα | erβ | ar |  | fshr | lhr | hmgra | hmgrb | star | cyp11a | 3βhsd | cyp17 | 17βhsd | cyp19a |
| **Brain** |  |  |  |  |  |  |  |  |  |  |  |  |  |  |  |  |  |  |  |  |  |  |  |  |
|  |  | gnrh2 | gnrh3 | gnrhr1 | gnrhr2 | gnrhr3 | gnrhr4 | fshb | lhb | cyp19b | era | erb | ar |  | fshr | lhr | hmgra | hmgrb | star | cyp11a | hsd3b | cyp17 | hsd17b | cyp19a |
| ghrh2 |  | 1.000 | 0.455 | 0.243 | **0.470** | -0.540 | 0.383 | 0.051 | **0.709** | 0.046 | 0.529 | 0.586 | 0.513 |  | **0.776** | 0.570 | -0.171 | 0.337 | 0.166 | 0.560 | 0.549 | 0.200 | **-0.823** | 0.242 |
|  |  |  | (0.077) | (0.402) | **(0.007)** | (0.070) | (0.144) | (0.864) | **(0.005)** | (0.867) | (0.043) | (0.017) | (0.042) |  | **(0.000)** | (0.021) | (0.577) | (0.239) | (0.538) | (0.024) | (0.042) | (0.457) | **(0.001)** | (0.449) |
| gnrh3 |  |  | 1.000 | 0.134 | 0.454 | -0.179 | 0.129 | -0.253 | 0.403 | 0.147 | 0.290 | 0.400 | 0.287 |  | 0.490 | 0.412 | -0.008 | 0.358 | 0.259 | 0.525 | 0.185 | -0.112 | -0.580 | 0.287 |
|  |  |  |  | (0.647) | (0.089) | (0.579) | (0.633) | (0.383) | (0.153) | (0.587) | (0.295) | (0.125) | (0.281) |  | (0.054) | (0.113) | (0.979) | (0.208) | (0.333) | (0.037) | (0.527) | (0.680) | (0.048) | (0.366) |
| gnrhr1 |  |  |  | 1.000 | 0.132 | -0.680 | **0.711** | -0.473 | 0.210 | 0.418 | 0.390 | 0.625 | 0.578 |  | 0.531 | -0.101 | 0.105 | 0.349 | 0.369 | 0.163 | -0.364 | -0.009 | -0.441 | 0.280 |
|  |  |  |  |  | (0.653) | (0.015) | **(0.004)** | (0.102) | (0.492) | (0.137) | (0.188) | (0.017) | (0.030) |  | (0.051) | (0.730) | (0.745) | (0.242) | (0.194) | (0.578) | (0.245) | (0.976) | (0.152) | (0.397) |
| gnrhr2 |  |  |  |  | 1.000 | -0.284 | 0.136 | 0.050 | 0.606 | 0.164 | 0.202 | 0.325 | 0.240 |  | 0.559 | 0.320 | 0.047 | 0.499 | 0.030 | 0.432 | 0.078 | -0.054 | -0.455 | 0.154 |
|  |  |  |  |  |  | (0.347) | (0.630) | (0.859) | (0.028) | (0.558) | (0.470) | (0.237) | (0.370) |  | (0.030) | (0.245) | (0.879) | (0.069) | (0.912) | (0.095) | (0.800) | (0.841) | (0.138) | (0.615) |
| gnrhr3 |  |  |  |  |  | 1.000 | -0.648 | 0.242 | -0.475 | -0.256 | -0.358 | **-0.865** | -0.525 |  | -0.495 | -0.240 | 0.442 | -0.235 | -0.528 | -0.431 | -0.002 | -0.272 | **0.781** | -0.426 |
|  |  |  |  |  |  |  | (0.023) | (0.426) | (0.119) | (0.422) | (0.230) | **(0.000)** | (0.065) |  | (0.102) | (0.452) | (0.150) | (0.463) | (0.064) | (0.141) | (0.996) | (0.368) | **(0.003)** | (0.146) |
| gnrhr4 |  |  |  |  |  |  | 1.000 | -0.157 | 0.249 | 0.564 | 0.449 | **0.705** | 0.390 |  | 0.430 | 0.055 | -0.333 | -0.024 | 0.369 | 0.200 | -0.029 | 0.419 | -0.587 | -0.203 |
|  |  |  |  |  |  |  |  | (0.576) | (0.391) | (0.018) | (0.081) | **(0.001)** | (0.109) |  | (0.097) | (0.829) | (0.266) | (0.935) | (0.145) | (0.441) | (0.919) | (0.094) | (0.045) | (0.527) |
| fshβ |  |  |  |  |  |  |  | 1.000 | 0.364 | 0.038 | 0.007 | 0.154 | -0.617 |  | -0.264 | -0.297 | -0.470 | -0.635 | -0.121 | -0.312 | 0.118 | 0.418 | 0.133 | -0.495 |
|  |  |  |  |  |  |  |  |  | (0.222) | (0.894) | (0.978) | (0.585) | (0.011) |  | (0.362) | (0.282) | (0.105) | (0.105) | (0.656) | (0.239) | (0.688) | (0.121) | (0.681) | (0.086) |
| lhβ |  |  |  |  |  |  |  |  | 1.000 | 0.123 | 0.168 | 0.641 | 0.082 |  | 0.636 | 0.189 | -0.295 | 0.193 | 0.244 | 0.353 | 0.362 | 0.449 | -0.681 | 0.165 |
|  |  |  |  |  |  |  |  |  |  | (0.674) | (0.567) | (0.014) | (0.782) |  | (0.014) | (0.519) | (0.351) | (0.528) | (0.401) | (0.215) | (0.224) | (0.107) | (0.015) | (0.609) |
| cyp19b |  |  |  |  |  |  |  |  |  | 1.000 | 0.434 | 0.403 | -0.004 |  | 0.090 | -0.229 | -0.374 | -0.358 | 0.216 | -0.111 | -0.496 | 0.112 | -0.281 | -0.652 |
|  |  |  |  |  |  |  |  |  |  |  | (0.093) | (0.108) | (0.989) |  | (0.741) | (0.377) | (0.208) | (0.208) | (0.405) | (0.671) | (0.060) | (0.681) | (0.375) | (0.022) |
| erα |  |  |  |  |  |  |  |  |  |  | 1.000 | 0.456 | 0.491 |  | 0.296 | 0.441 | -0.249 | -0.129 | 0.138 | 0.128 | 0.150 | -0.010 | -0.438 | -0.024 |
|  |  |  |  |  |  |  |  |  |  |  |  | (0.066) | (0.039) |  | (0.266) | (0.077) | (0.413) | (0.661) | (0.585) | (0.612) | (0.580) | (0.971) | (0.154) | (0.939) |
| erβ |  |  |  |  |  |  |  |  |  |  |  | 1.000 | 0.285 |  | **0.661** | 0.162 | -0.497 | -0.148 | **0.682** | **0.660** | 0.397 | 0.568 | **-0.822** | 0.358 |
|  |  |  |  |  |  |  |  |  |  |  |  |  | (0.224) |  | **(0.004)** | (0.494) | (0.084) | (0.614) | **(0.002)** | **(0.003)** | (0.114) | (0.014) | **(0.001)** | (0.254) |
| ar |  |  |  |  |  |  |  |  |  |  |  |  | 1.000 |  | 0.564 | **0.593** | 0.060 | 0.489 | 0.350 | 0.506 | 0.277 | 0.039 | **-0.780** | 0.541 |
|  |  |  |  |  |  |  |  |  |  |  |  |  |  |  | (0.015) | **(0.005)** | (0.845) | (0.076) | (0.130) | (0.023) | (0.283) | (0.871) | **(0.003)** | (0.056) |
| **Gonad** |  |  |  |  |  |  |  |  |  |  |  |  |  |  |  |  |  |  |  |  |  |  |  |  |
| fshr |  |  |  |  |  |  |  |  |  |  |  |  |  |  | 1.000 | 0.549 | -0.118 | 0.369 | **0.661** | **0.809** | 0.523 | 0.519 | **-0.806** | 0.443 |
|  |  |  |  |  |  |  |  |  |  |  |  |  |  |  |  | (0.018) | (0.702) | (0.194) | **(0.003)** | **(0.000)** | (0.045) | (0.027) | **(0.002)** | (0.149) |
| lhr |  |  |  |  |  |  |  |  |  |  |  |  |  |  |  | 1.000 | 0.023 | 0.368 | 0.210 | **0.665** | **0.612** | 0.259 | -0.516 | 0.630 |
|  |  |  |  |  |  |  |  |  |  |  |  |  |  |  |  |  | (0.940) | (0.195) | (0.389) | **(0.002)** | **(0.009)** | (0.285) | (0.086) | (0.028) |
| hmgra |  |  |  |  |  |  |  |  |  |  |  |  |  |  |  |  | 1.000 | **0.770** | -0.402 | -0.298 | -0.266 | -0.660 | 0.265 | 0.396 |
|  |  |  |  |  |  |  |  |  |  |  |  |  |  |  |  |  |  | **(0.002)** | (0.173) | (0.322) | (0.380) | (0.014) | (0..405) | (0.202) |
| hmgrb |  |  |  |  |  |  |  |  |  |  |  |  |  |  |  |  |  | 1.000 | -0.105 | 0.170 | -0.006 | -0.389 | -0.149 | 0.664 |
|  |  |  |  |  |  |  |  |  |  |  |  |  |  |  |  |  |  |  | (0.722) | (0.562) | (0.985) | (0.169) | (0.645) | (0.019) |
| star |  |  |  |  |  |  |  |  |  |  |  |  |  |  |  |  |  |  | 1.000 | **0.652** | 0.265 | 0.499 | -0.459 | 0.484 |
|  |  |  |  |  |  |  |  |  |  |  |  |  |  |  |  |  |  |  |  | **(0.002)** | (0.321) | (0.030) | (0.134) | (0.094) |
| cyp11a |  |  |  |  |  |  |  |  |  |  |  |  |  |  |  |  |  |  |  | 1.000 | **0.773** | **0.689** | -0.673 | 0.596 |
|  |  |  |  |  |  |  |  |  |  |  |  |  |  |  |  |  |  |  |  |  | **(0.000)** | **(0.001)** | (0.016) | (0.031) |
| 3βhsd |  |  |  |  |  |  |  |  |  |  |  |  |  |  |  |  |  |  |  |  | 1.000 | **0.688** | -0.401 | 0.403 |
|  |  |  |  |  |  |  |  |  |  |  |  |  |  |  |  |  |  |  |  |  |  | **(0.005)** | (0.196) | (0.194) |
| cyp17 |  |  |  |  |  |  |  |  |  |  |  |  |  |  |  |  |  |  |  |  |  | 1.000 | -0.417 | 0.010 |
|  |  |  |  |  |  |  |  |  |  |  |  |  |  |  |  |  |  |  |  |  |  |  | (0.177) | (0.975) |
| 17βhsd |  |  |  |  |  |  |  |  |  |  |  |  |  |  |  |  |  |  |  |  |  |  | 1.000 | -0.308 |
|  |  |  |  |  |  |  |  |  |  |  |  |  |  |  |  |  |  |  |  |  |  |  |  | (0.330) |
| cyp19a |  |  |  |  |  |  |  |  |  |  |  |  |  |  |  |  |  |  |  |  |  |  |  | 1.000 |

The values in parentheses are *p* values.

**Table S6.** Result of principal component analysis

|  |  | Females | |  | Males | |
| --- | --- | --- | --- | --- | --- | --- |
|  |  | PC1 | PC2 |  | PC1 | PC2 |
| Eigenvalues |  | 10.86 | 3.90 |  | 8.73 | 4.03 |
| Variance (%) |  | 49.36 | 17.71 |  | 39.68 | 18.32 |
| Accumulative (%) |  | 48.36 | 67.07 |  | 39.68 | 58.00 |
| Factor loadings |  |  |  |  |  |  |
| *ghrh2* |  | **0.80** | -0.45 |  | **0.88** | -0.25 |
| *gnrh3* |  | 0.24 | 0.79 |  | 0.60 | 0.35 |
| *gnrhr1* |  | 0.18 | -0.27 |  | 0.39 | 0.59 |
| *gnrhr2* |  | 0.19 | -0.60 |  | 0.65 | 0.12 |
| *gnrhr3* |  | **-0.87** | 0.10 |  | -0.70 | -0.02 |
| *gnrhr4* |  | 0.60 | 0.75 |  | 0.19 | -0.03 |
| *fshβ* |  | -0.36 | -0.25 |  | -0.08 | **-0.90** |
| *lhβ* |  | 0.70 | 0.09 |  | **0.99** | -0.35 |
| *cyp19b* |  | -0.78 | 0.53 |  | -0.01 | -0.06 |
| *erα* |  | -0.71 | -0.26 |  | 0.44 | -0.07 |
| *erβ* |  | -0.22 | **0.86** |  | **0.84** | -0.16 |
| *ar* |  | **0.92** | 0.14 |  | 0.72 | 0.48 |
| *fshr* |  | -0.73 | 0.44 |  | **0.92** | 0.09 |
| *lhr* |  | **0.89** | -0.06 |  | 0.71 | 0.11 |
| *hmgra* |  | **0.87** | 0.03 |  | -0.31 | **0.80** |
| *hmgrb* |  | **0.94** | -0.20 |  | 0.19 | **0.85** |
| *star* |  | -0.77 | -0.04 |  | 0.61 | -0.09 |
| *cyp11a* |  | -0.60 | -0.34 |  | **0.89** | -0.08 |
| *3βhsd* |  | 0.79 | 0.58 |  | 0.68 | -0.38 |
| *cyp17* |  | -0.29 | -0.27 |  | 0.57 | -0.67 |
| *17βhsd* |  | -0.64 | -0.32 |  | **-0.89** | -0.11 |
| *cyp19a* |  | **-0.82** | 0.19 |  | 0.52 | 0.53 |

Values ≥ 0.80 for female and male zebrafish are shown in bold.
